# Sudden Sensorineural Hearing Loss as the Presenting Symptom of Varicella Zoster Virus Rhombencephalitis: A Case Report

**DOI:** 10.7759/cureus.100632

**Published:** 2026-01-02

**Authors:** Christian Chess, Laura Bateman, Chun Pang, Nicola Giffin, Louise Melia

**Affiliations:** 1 Otolaryngology, Royal United Hospital, Bath, GBR; 2 Radiology, Royal United Hospital, Bath, GBR; 3 Neurology, Royal United Hospital, Bath, GBR

**Keywords:** clinical case report, clinical neurotology, herpes zoster virus, mri images, sudden sensorineural hearing loss (ssnhl), varicella zoster virus encephalitis

## Abstract

Sudden sensorineural hearing loss (SNHL) is an ENT emergency that requires prompt diagnosis and treatment. Most cases are thought to be idiopathic, and early oral steroid initiation is thought to increase the likelihood of recovery. MRI of the internal auditory meatus (IAM) is the imaging modality of choice, as retrocochlear pathology needs to be excluded. We report the case of a 54-year-old man who presented with left-sided sudden SNHL in the absence of any other symptoms. MRI IAM demonstrated inflammation in the posterior fossa (rhombencephalitis), prompting an urgent neurology referral. Lumbar puncture detected varicella zoster virus (VZV) encephalitis, and the patient was treated with intravenous acyclovir. He subsequently developed a vesicular rash weeks later. Follow-up MRI showed near-complete lesion resolution, and his hearing showed minor improvement. This case demonstrates how expedient MRI can detect unusual causes of sudden SNHL, enabling timely management and increasing the likelihood of resolution.

## Introduction

Sudden sensorineural hearing loss (SNHL) is a common ENT emergency, defined as a hearing loss of 30 dB or greater over three contiguous frequencies occurring over a 72-hour period [1]. Potential causative agents include viral or bacterial infections, trauma, vascular incidents, or autoimmune conditions, but most cases are idiopathic [2].

Varicella zoster virus (VZV) is a highly contagious neurotropic human herpes virus. It causes two clinically distinct diseases: varicella and herpes zoster [3]. Varicella (chickenpox) is a common childhood infection that typically presents with fever, malaise, and a widespread vesicular rash [4]. Following primary infection, VZV becomes latent within the dorsal root and cranial root ganglia; subsequent reactivation of the virus (often triggered by immunocompromise, immunosuppression, or stress) can lead to herpes zoster (shingles). This typically manifests as a burning or stinging neuropathic pain in a dermatomal distribution, followed by a vesicular rash.

VZV reactivation can cause a wide range of serious neurological and systemic symptoms, including vasculopathy, encephalitis, myelitis, aseptic meningitis, and cranial polyneuropathies due to the neurotropic nature of the virus [5,6]. Cranial nerve involvement can result in auditory and vestibular symptoms; Ramsay Hunt syndrome is well recognised for causing facial nerve palsy, vertigo, and hearing loss.

Although SNHL is a less commonly reported complication of VZV, it can have a profound impact on a patient’s quality of life. Reactivation of VZV in the vestibulocochlear nerve (CN VIII) can lead to SNHL through multiple mechanisms, including direct viral-induced neuritis, vasculopathy-induced ischaemia, and immune-mediated cochlear damage.

Hearing impairment in patients with herpes zoster infections is mild to moderate in most cases, with either cochlear or retrocochlear involvement; 54% of hearing loss cases occur at high frequencies, whereas only 19% affect speech frequencies [7,8]. Of individuals with cranial nerve injury due to herpes zoster, 10-25% experience some degree of hearing loss. This can range in severity from mild to profound, affecting either specific frequencies or resulting in total bilateral SNHL [9].

Diagnosis of VZV-related conditions relies on detecting VZV DNA via polymerase chain reaction (PCR) or the presence of anti-VZV IgG antibodies in CSF or peripheral blood mononuclear cells [10]. Treatment consists of a two-week course of intravenous aciclovir, with or without adjunctive corticosteroids. Initiation of treatment within 72 hours of symptom onset has been associated with improved auditory outcomes.

VZV reactivation can cause neurological disease in the absence of a vesicular rash (zoster sine herpete), including meningoencephalitis and cranial nerve palsies. Vestibulocochlear involvement without rash appears rare and is mainly supported by isolated case reports [11].

To our knowledge, no published cases have reported VZV-associated SNHL occurring in isolation, without other neurological or dermatological symptoms at the time of symptom onset. This highlights the need to consider VZV in unexplained SNHL, even in the absence of classic features such as a vesicular rash.

## Case presentation

A 54-year-old male patient presented with a sudden loss of hearing in his left ear upon waking on December 24. He had no associated otalgia, aural fullness, rotational vertigo, or headaches. Two weeks before the onset of his hearing loss, he had an upper respiratory tract infection with associated fever, lethargy, and malaise, but had recovered within a few days with no lasting symptoms. Although he specifically denied dizziness or light-headedness, he mentioned that he had felt mildly unsteady on his feet since his upper respiratory tract infection.

His past medical history was otherwise unremarkable. He had completed his childhood vaccination schedule and had primary varicella (chickenpox) infection in childhood, from which he made a full recovery. To his knowledge, he had never suffered from an outbreak of shingles or VZV reactivation and had never been vaccinated against the disease. The patient had previously been diagnosed with mild left-sided SNHL and bilateral tinnitus, which had been fully investigated via MRI several years before and found to be idiopathic. He was already a bilateral hearing aid user, and so initially thought his hearing aid was malfunctioning. He was particularly concerned about the sudden hearing loss, as he already considered his hearing to be poor, and the potential impact on his livelihood.

When symptoms persisted, he sought advice from his general practitioner (GP). Immediately after this initial consultation, the patient was initiated on high-dose oral steroids (prednisolone 60 mg once daily for 10 days with no tapering). These steroids were started three days after the initial onset of hearing loss. An urgent referral was sent to ENT, who saw the patient on January 4.

**Figure 1:**
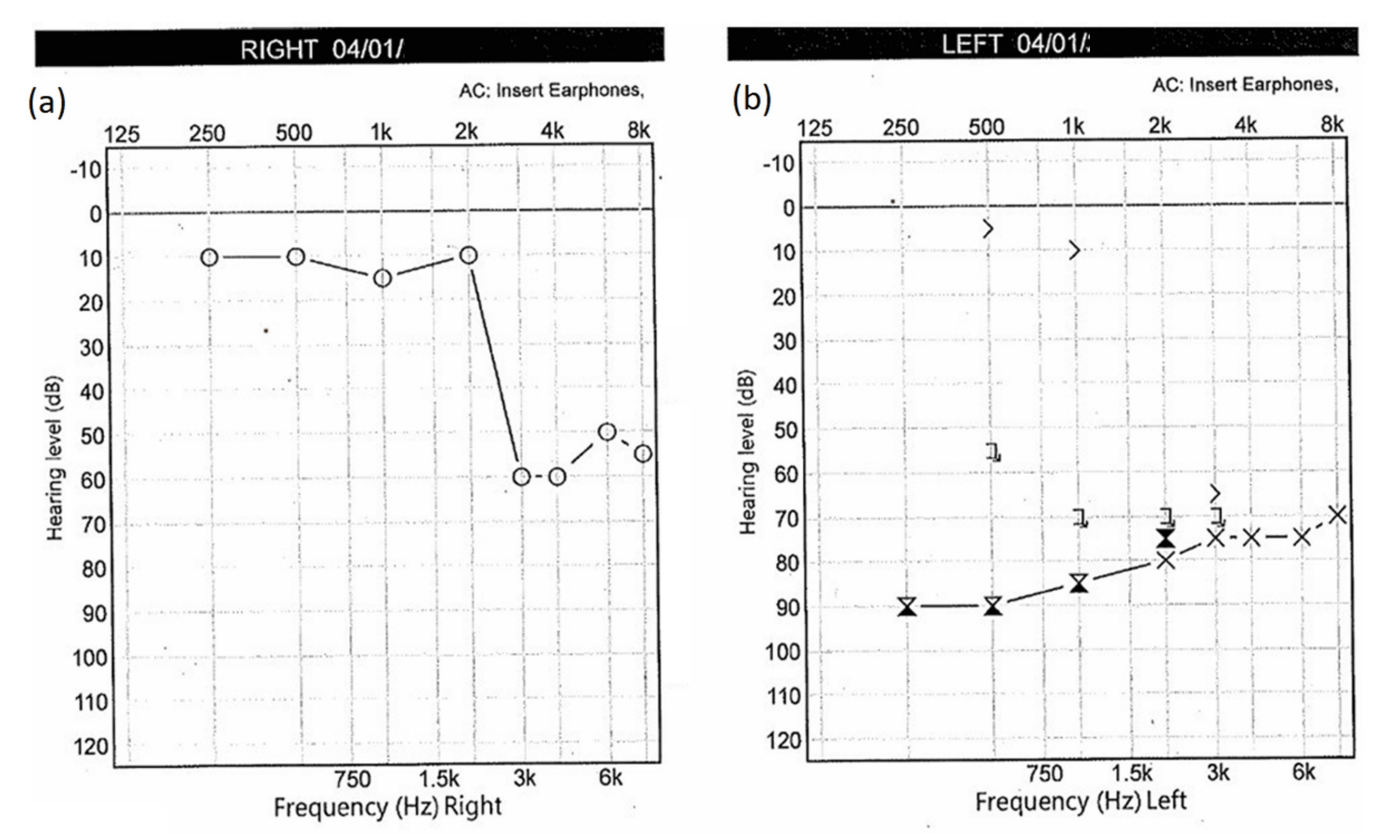
Pure tone audiogram at presentation. (a) The right ear shows a known high pitch (3kHz +) sensorineural hearing loss. (b) Audiogram showing a new profound sensorineural hearing loss in left ear across all frequencies.

Otoscopy was normal. There was no evidence of cutaneous vesicular rash or oral mucosal lesions. Pure tone audiometry showed a left-sided hearing loss of approximately 30+ decibels over four frequencies. Tympanometry was Type A (Figure 1). 

**Figure 2:**
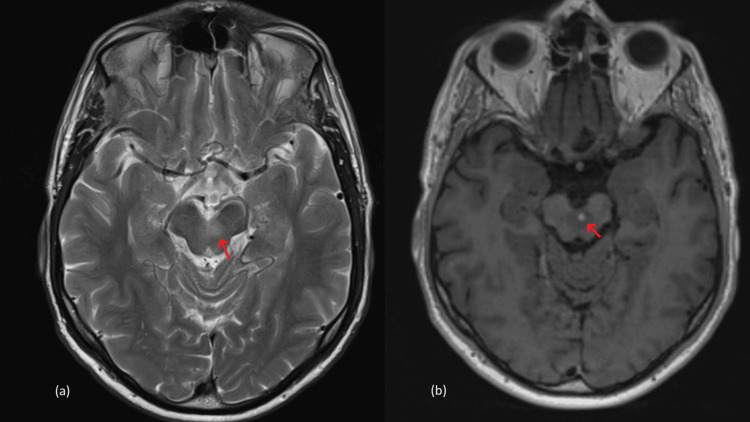
MRI IAM images (a) T2 weighted axial sequence, (b) T1 space post contrast axial sequence. Internal auditory meatus (IAM) magnetic resonance image (MRI). (a) Axial image through the ventral midbrain showing left oedema. (b) Axial MRI selected section through the ventral midbrain showing an enhancing nodule indicating left rhombencephalitis. Both areas of interest have been circled.

**Figure 3:**
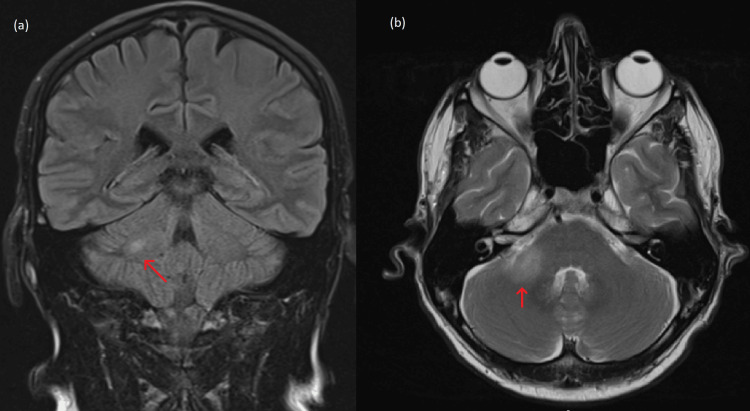
MRI whole brain. (a) FLAIR coronal sequence. (b) T2 post contrast axial sequence. Magnetic resonance imaging (MRI) of whole brain showing enhancement in the cerebellar peduncle. (a) Selected section through the right middle cerebellar peduncle showing faint oedema. (b) Selected section though the cerebellar peduncle showing an enhancing right nodule indicating a second focus of rhombencephalitis. FLAIR: fluid-attenuated inversion recovery

After repeating his examination, a non-contrast MRI of the internal auditory meatus (IAM) was arranged. The MRI highlighted two lesions in the posterior aspect of the cranial fossa, specifically, in the right cerebellar tonsil and the pons. A dedicated MRI with contrast was conducted on January 26, which showed high T2 signal lesions 14 mm x 20 mm within the right cerebellar peduncle and another smaller lesion in the left mid-brain, in keeping with rhombencephalitis (Figures 2, 3).

The patient was assessed on February 15 by Neurology, and a detailed neurological examination was conducted. Gait was normal, tandem gait was wobbly, and Romberg’s test leaned to the left. A lumbar puncture was conducted, and blood tests were done, which were broadly normal, while CSF PCR showed VZV only (Tables 1, 2).

**Table 1:** Serum blood results at time of admission. Serum biochemistry and haematology results on admission for treatment of varicella zoster virus (VZV) rhomboencephalitis. The results show broadly normal blood results, with no clear indicator of viral or bacterial infection. As a side note, erythrocyte sedimentation rate (ESR) and plasma viscosity were not conducted.

Laboratory Test	Reference Ranges	Test Result
Biochemistry		
C-Reactive Protein	< 5 mg/L	1
Sodium	133-146 mmol/L	139
Potassium	3.5-4.0 mmol/L	3.9
Creatinine	64-104 mcmol/L	94
Estimated Glomerular Filtration Rate	>90 ml/minute/1.73m2	79
Haematology		
Total White Cell Count	4.0-11.0x10^9^/L	6.7
Red Blood Cell count	4.50-6.00x10^12^/L	4.65
Haemaglobin	130-170 g/L	141
Haematocrit	0.400-0.520 L/L	0.427
Mean Cell Volume	83.0-100.0 fL	91.9
Platelets	150-450x10^9^/L	186
Neutrophils	1.5-8.0x10^9^/L	4.2
Lymphocytes	1.0-4.0x10^8^/L	1.8
Monocytes	0.20-1.00x10^9^/L	0.54
Eosinophils	≤0.51x10^9^/L	0.17
Basophils	≤0.2x10^9^/L	0.07

**Table 2:** Cerebral spinal fluid (CSF) from a lumbar puncture investigated via biofire polymerase chain reaction (PCR) assay array. Varicella zoster virus (VZV) was highlighted as positive, ultimately leading to the diagnosis of VZV rhomboencephalitis.

PCR test	Result
*Escherichia coli* K1	Negative
Haemophilus influenzae	Negative
Listeria monoctogenes	Negative
Neisseria meningitidis	Negative
Streptococcus agalactiae	Negative
Streptococcus pneumoniae	Negative
Cytomegalovirus (CMV)	Negative
Enterovirus	Negative
Herpes simplex 1 (HSV-1)	Negative
Herpes simplex 2 (HSV-2)	Negative
Human herpes virus 6 (HHV-6)	Negative
Human parechovirus (HPV)	Negative
Varicella zoster virus (VZV)	POSITIVE
Cryptococcus neoformans/gattii	Negative

The patient was admitted for intravenous aciclovir (700 mg three times a day for two weeks), which was commenced on March 4, 57 days after the conclusion of the oral steroid therapy. After 12 days of anti-viral treatment, the patient then developed a vesicular rash over his right mid-torso, 83 days after the initial complaint of reduced hearing. He was given paracetamol and amitriptyline for analgesia. A second lumbar puncture was performed on March 19 after treatment completion, which was negative for VZV.

**Figure 4:**
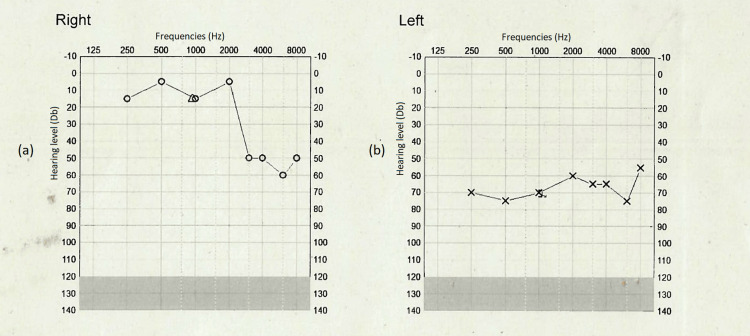
Pure tone audiogram (PTA) post treatment. (a) Right ear. (b) Left ear. Audiogram with a small improvement post treatment with antivirals (aciclovir). (a) Right sided hearing remained poor from a previously known sensorineural hearing loss. (b) Left side hearing improved by at least 20 decibels in most frequencies post treatment.

A repeat MRI with contrast scan was arranged for May 4, seven weeks post-IV antiviral treatment. This confirmed that there had been near total resolution of the inflammatory change. The patient was seen in the clinic ENT again with pure tone audiometry showing a minor improvement in hearing (Figure 4).

## Discussion

To our knowledge, this is the first reported case of VZV reactivation causing SNHL in isolation, without other neurological or dermatological symptoms at the time of presentation. Hearing loss associated with VZV is well recognised, first described by James Ramsey Hunt in 1907 [12]. Since then, VZV has been linked to encephalitis, vasculopathy, segmental motor weakness, myelitis, cranial nerve syndromes, Guillain-Barré syndrome, meningoencephalitis, and, more recently, giant cell arteritis [6]. Despite this, isolated SNHL as the sole presenting feature of VZV reactivation likely remains an under-recognised entity.

Shao et al. described a case of sudden SNHL associated with primary VZV infection [13]. Schwab et al. described a similar case of transient SNHL in a child due to a VZV meningitis [14]. The patient was treated with aciclovir and made a full recovery. Interestingly, the child was vaccinated (single-antigen live attenuated) only 18 months prior to the onset of symptoms. Hearing loss is not routinely tested as a potential consequence of VZV infection in children, and its true incidence may be underestimated.

Zoster sine herpete (ZSH), where VZV-induced neurological symptoms can occur without a rash, can affect cranial nerves, as well as peripheral nerves [15-17]. Ultimately, more research on ZSH, especially epidemiological surveys and the development of guidelines for diagnosis and treatment are needed, as most existing literature consists of case reports [18].

Rhombencephalitis has a broad range of differentials, with the most common bacterial cause being Listeria, and viral causes include Epstein-Barr virus (EBV), herpes simplex virus (HSV), cytomegalovirus (CMV), and VZV. The absence of additional symptoms, such as a rash, may lead to underrecognition of VZV as a cause of rhombencephalitis.

ENT UK (London, United Kingdom) guidance for suspected idiopathic sudden SNHL recommends prompt steroid treatment and arranging a routine MRI of the IAM to exclude retrocochlear pathology [19]. However, relying on a routine MRI pathway alone risks delaying investigation and treatment when the cause is not idiopathic and time-critical diagnoses are possible.

In this case, early MRI played a crucial role in detecting the underlying pathology. Without an urgent MRI following initial ENT referral, this patient is likely to have experienced significant delays in specialist neurology input. The potential progression of this patient's VZV infection, had it not been promptly diagnosed, remains uncertain, but the possibility of further neurological deterioration is a distinct concern. Interestingly, the only time this patient exhibited any rash-like symptoms was 12 days into IV acyclovir treatment: nearly three months after the initial onset of hearing loss. This suggests that VZV-associated SNHL may present insidiously, and there may be a strong justification for prioritising MRI scans in cases of SNHL to identify atypical causes.

Zoster vaccination had no role in the acute treatment of this case’s VZV episode. Following herpes zoster, VZV-specific cell-mediated immunity is naturally boosted and may persist for at least two years [20]. Accordingly, ENT UK guidance notes that the benefit of offering the zoster vaccine immediately after recovery is unclear and recommends deferring vaccination until symptoms have ceased. After full clinical recovery, the recombinant zoster vaccine could be considered to reduce future reactivation risk. However, at age 55, it is usually NHS-eligible only for those who are severely immunosuppressed.

The approach of early and urgent MRI must be carefully balanced against resource availability, cost, and the expertise required to interpret results. It remains to be determined whether the potential benefits of identifying additional cases justify changes to current imaging protocols.

## Conclusions

Hearing loss can be devastating to a patient’s quality of life, with social, financial, and wider health implications, and early diagnosis and treatment can significantly improve outcomes. This case highlights the need for increased clinical suspicion of VZV (even in the absence of rash) in unexplained SNHL, as early detection and treatment may prevent further neurological complications. Patients with unexplained or atypical hearing loss may benefit from an early MRI and CSF virology testing to detect rarer causes.
